# Development and implementation of an LGBT initiative at a health sciences library: the first eighteen months

**DOI:** 10.5195/jmla.2019.422

**Published:** 2019-10-01

**Authors:** Jessica Petrey

**Affiliations:** Kornhauser Health Sciences Library, University of Louisville, Louisville, KY, jlpetr04@louisville.edu

## Abstract

**Background:**

The University of Louisville School of Medicine is the pilot site for the eQuality project, an initiative to integrate training for providing care to lesbian, gay, bisexual, and transgender (LGBT) patients into the standard medical school curriculum. Inspired by and in support of this School of Medicine initiative, Kornhauser Health Sciences Library staff have developing our own initiative. Because of past and current lack of competent provider training and the resulting need for patients to be knowledgeable self-advocates, however, our initiative was broadened to include the goal of providing LGBT individuals in our communities—both on campus and in the broader public—with the resources and tools that they need to access information about their own health.

**Case Presentation:**

This paper describes the development of that twofold initiative and the tangible methods used in its implementation, including collection development, interdepartmental collaboration, electronic resource guide creation, and community engagement through outreach.

**Conclusions:**

Outcomes of the initiative to date will also be discussed, along with plans for further development.

## BACKGROUND

Multiple studies evaluating the health information needs of lesbian, gay, bisexual, and transgender (LGBT) people [1–3] and describing the health disparities of this population [4–6] have been published, and discussions are taking place among health sciences librarians about these needs and disparities, and librarians’ role in providing culturally competent services to LGBT patrons [7, 8]. Also well documented is a lack of health care provider awareness of and comfort with treating LGBT patients [9–11]. This report describes the implementation of an initiative in a health sciences library that works both to provide better services to LGBT patrons and to support the cultural competence training of health professionals who will care for them.

The University of Louisville (UofL) is the only school in Kentucky and in the South to receive a perfect score from the Campus Pride Index, designating it as an LGBT friendly campus according to a variety of metrics including student life, academics, institutional commitment, and health [12]. The UofL School of Medicine is the pilot site for the eQuality project, an initiative to integrate training for providing care to LGBT patients into the standard medical school curriculum. The eQuality project was recognized with Louisville Business First’s Healthcare Heroes award for its work in preparing medical students to better care for LGBT patients [13] and won the 2016–2017 Innovation in Medical Education Award from the Southern Group on Educational Affairs [14].

Inspired by and in support of the School of Medicine project, the author began developing an initiative at the UofL Kornhauser Health Sciences Library in March 2016. Due to past and current lack of competent provider training [9–11] and the resulting need for patients to be knowledgeable self-advocates, the library’s initiative was broadened to include the secondary goal of providing LGBT individuals in our communities—both on campus and in the broader public—with resources and tools to access information about their health. This twofold mission has manifested itself in the form of faculty and staff training, evaluation and development of collections, the presence of a designated LGBT reference librarian, electronic resource guide creation, community engagement, and outreach.

## CASE PRESENTATION

To ensure the library’s goals were compatible with the mission of the School of Medicine eQuality project, a meeting was arranged with the Health Sciences Campus (HSC) LGBT Center director and staff. The background for the eQuality project, educational programs, other services offered by the HSC LGBT Center, and the staff’s most-referenced resources were discussed. This meeting also made the HSC LGBT Center staff aware of the library’s interest in allyship to enable future opportunities for collaboration. The primary actionable result of this meeting was a prioritization of training for library faculty and staff to provide foundational knowledge from which to develop the rest of the initiative.

### Training

Safe Zone training is a curriculum developed and delivered by HSC LGBT Center staff to empower groups and organizations on campus to create safe academic or health care spaces for each other and the people they serve. To ensure a standardized understanding of the LGBT community and level of service, one such training was arranged and adapted for library faculty and staff. This allowed library workers to gain an understanding of LGBT vocabulary, health disparities, and stigma through a traditional lecture from the HSC LGBT Center director and a panel of LGBT community members.

Of the seventeen library faculty and staff, fourteen were available and attended the training. While primarily an educational opportunity, this training was also an opportunity to complete a visible act confirming the library’s interest in and support for the LGBT community, and all participants received ally stickers to display in their workstations. The likelihood that an LGBT patron would approach and trust library staff with their information-seeking needs is improved by these tangible demonstrations of support for the community [1, 3].

To supplement this baseline level of knowledge and competencies needed for all library faculty and staff, the author sought additional educational opportunities to better serve this population and their care providers. The Louisville Lesbian, Gay, Bisexual, Transgender and Queer (LGBTQ) Health Summit—hosted by the UofL School of Medicine Office of Undergraduate Medical Education, Office for Community Engagement and Diversity, and HSC LGBT Center—was a daylong retreat of educational and collaborative opportunities. Sessions included panels of LGBT community members sharing experiences, lectures on defining health disparities, and small group discussion geared toward developing ideas for actionable steps for improving the health of the local LGBT community.

The Louisville Pre-Exposure Prophylaxis (PrEP) Summit was a collaborative effort hosted by the Louisville Metro Public Health Department, UofL School of Medicine, and the Office of Diversity & Inclusion to educate local health professionals, educators, and the public on the use of medication to reduce the chance of patients acquiring the human immunodeficiency virus (HIV). This daylong series of lectures and group activities was free and included information on LGBT populations, with most of the sessions focused more generally on the use of PrEP, administration guidelines, and insurance or payment navigation. While both were valuable educational and networking opportunities, the population-specific nature of the LGBTQ health summit was a more efficient use of the librarian’s time.

The most extensive and impactful of these trainings has been the LGBT Competency Certificate series, from which the author graduated in April 2017. Registration for this series of classes, hosted and organized by the UofL HSC LGBT Center, was open to faculty, staff, and students at the Schools of Nursing, Dentistry, Medicine, and Public Health, and the author was the only librarian to attend. The courses in this series varied in format, including lectures, panels, and discussions. Topics also varied, including medical and legal disparities affecting LGBT patients, anecdotal accounts of LGBT experiences with the health care system, medical implications of prolonged cross-sex hormone therapy, sexual history taking, and creation of a welcoming environment to improve health outcomes. In addition to the data, statistics, and clinical training received, the librarian’s exposure to the kinds of questions that other attendees were asking during each LGBT Competency Certificate session guided the librarian’s thought process toward the types of resources that librarians could gather and make available to patrons.

### Collections

In addition to resources mentioned in the meeting with the HSC LGBT Center staff and in training sessions, the author conducted searches in OCLC WorldCat, Rittenhouse Book Distributors, and Amazon.com to evaluate the available LGBT health literature. Most of the items with a focus on LGBT health that was retrieved in these searches took the form of self-help books, memoirs, anthologies, magazines, and narrative nonfiction. In conjunction with collection librarians, the author decided to continue excluding these types of items from the library’s collection. Publications by Flemming [15], McKay [4], and Phillips [16] containing annotated lists of LGBT information resources were also consulted. The items that ultimately were recommended for purchase or subscription were clinically based textbooks or journals, and twelve books and five journals are now featured in Kornhauser’s LGBT collection.

Exceptions were made, however, for publications and free resources from professional organizations such as the Fenway Institute, Centers for Disease Control and Prevention (CDC), Substance Abuse and Mental Health Services Administration (SAMHSA), and the Gay and Lesbian Medical Association (GLMA). From these organizations, the author gathered guidelines, pamphlets, flyers, and handouts containing information for current or trainee providers, frontline office staff, and patients.

### Resource guides

An online resource guide was conceptualized from the beginning of the initiative to ensure that the paid and free resources that had been gathered were more accessible to patrons who had a preference for electronic information seeking [2, 17]. As resources were collected, however, it became clear that a division by audience would be beneficial: Community members would likely be less interested in textbooks and professional journals and, more importantly, would not have off-campus access to these items. Thus, two separate guides were developed: a consumer health–level guide featuring free resources and links to provider directories (LGBT Health & Library Resources) and a provider-level guide providing access to library resources, patient handouts, and free resources containing information on creating a more welcoming practice and better patient experience (Competent Care for LGBT and DSD individuals).

Both guides were developed using Springshare’s LibGuides, an electronic content management system for library websites. In addition to the variety of audience-specific resources that these online guides provide access to, they allow visitors to contact an LGBT-friendly librarian by email, by phone, or anonymously through a chat box. These guides are linked on the library website, discoverable through Google, and promoted through campus channels, social media, and outreach events. The consumer health–level guide has been visited 904 times in the 3 years since its launch in April 2016, and the provider-level guide has been visited 601 times since its launch in August 2016.

### Inclusive restrooms

To make the library a more welcoming physical space, the multi-stall restrooms have been designated as gender-inclusive. Design and wording have been strategically chosen to emphasize that although the restrooms still have male and female designations, all are welcome to use them regardless of gender identity or expression. These signs also direct patrons to the closest gender-neutral restrooms on campus that have a single stall with a locking door. The university LGBT Center maintains a list of all gender-neutral restrooms located on all campuses, and the website for the list is included at the bottom of the signs.

### Promotion and outreach

Librarians and staff have shared information and resources through community engagement at local outreach events. Promotional flyers were designed for both guides, as was a pamphlet on finding quality LGBT health information online. GLMA’s “Ten Things…” lists, provider directory brochures, and a UofL Physicians flyer on LGBTQ care were provided. The library also contributed materials to the resource tables at the PrEP and LGBTQ Health Summits. Tabling at the HSC Pride Cookout and the Kentucky Association of LGBTQ Higher Education’s Come Together Kentucky Conference, an annual conference for Kentucky LGBT college students, provided visibility for the library initiative’s efforts, a platform to promote our online resource guides, and an opportunity for the campus and statewide community to gather resources from the table. Librarians were available at both events to discuss positive health information-seeking strategies with attendees.

The core outreach event of this initiative has been the Louisville Pride Festival, at which the library has set up a nonprofit booth annually since 2016. Library staff distributed LGBT health information, promoted library resources and services, and talked with the community about their needs. Rainbow-colored, brain-shaped stress balls, presenting a health focus while remaining festive, were purchased, printed with our consumer health LibGuide uniform resource locator (URL), and given away as promotional items.

During the 2016 event, the librarian and staff reported interacting with 211 community members and had 263 more visitors to the booth who took materials but did not speak with anyone, for a total of 474 community members reached. At the 2017 event, meaningful interactions increased to 278 and visits-only increased to 294, for a total of 572 community members reached. In 2018, however, engagement dropped to 217 interactions and 138 visits, presumably due to poor weather. Promotion and outreach should take place only after trainings have been conducted, spaces have been evaluated, and collections of resources have been gathered to ensure that library staff and collections are prepared to support any information seeking that occurs as a result of such activities.

## DISCUSSION

As a direct result of the event attendance and networking that has taken place at various events, flyers advertising our consumer health guide are spreading outside the campus community and are even hanging in the exam rooms of a local clinic that has no affiliation with the university. We also have had clinical faculty buy-in, with departments sharing the consumer health resource guide with their LGBT patients during clinic appointments and through flyers in waiting rooms. Event and class attendance also provided the opportunity to network with faculty and students to promote our LGBT liaison librarian and literature searching services on LGBT reference questions.

The least tangible but arguably most important result of this initiative has been the goodwill generated from the visible, active allyship on our campus and in the broader community, as Fikar [1] and Morris [3] both found to be essential to LGBT information seeking. Community patrons might have come in because they have seen flyers and chosen the library as a safe place to research their health. HSC students who are themselves LGBT might have felt more comfortable entering the library and interacting with our staff because they noticed ally stickers, flyers, or the library’s participation in campus Pride events. Patients may have received better care because their health care providers have used our resources to educate themselves on the needs of this patient population, because this education reduces knowledge gaps contributing to the disease burden and health disparities that Aguilar [9], Blondeel [5], White [10], and Williams [11] described.

What started as an effort to support a new curriculum in training health care professionals to care for LGBT patients evolved to also better serve the LGBT patients themselves and the campus and local LGBT community, and the idea of evolution has been a recurring motif throughout this initiative. Planning one resource guide evolved into creating two; educational opportunities became opportunities to educate; and research into clinical textbook availability resulted in a collection of pamphlets, handouts, and brochures.

### Future plans

A second round of Safe Zone training for new hires and those who were unable to attend the first session will be offered. The library hopes to expand outreach efforts to a larger regional Pride Festival, and further curation of the online resource guides and collections are planned for the future of this initiative, while accepting that the flexibility to adjust plans if necessary and the proactivity to seek out and meet needs that have not yet revealed themselves are integral to the success and mission of this initiative. The author also plans to investigate and, if necessary, develop methods for formal evaluation of this initiative’s success.

## 

**Jessica Petrey**, jlpetr04@louisville.edu, Kornhauser Health Sciences Library, University of Louisville, Louisville, KY
